# Perception of Caregiving During Childhood is Related to Later Executive Functions and Antisocial Behavior in At-Risk Boys

**DOI:** 10.3389/fpsyt.2020.00037

**Published:** 2020-02-06

**Authors:** Anna Harwood-Gross, Bar Lambez, Ruth Feldman, Yuri Rassovsky

**Affiliations:** ^1^Department of Psychology, Bar-Ilan University, Ramat-Gan, Israel; ^2^Center for Developmental, Social, and Relationship Neuroscience, Interdisciplinary Center, Herzliya, Israel; ^3^Leslie and Susan Gonda (Goldschmied) Multidisciplinary Brain Research Center, Bar-Ilan University, Ramat-Gan, Israel; ^4^Department of Psychiatry and Biobehavioral Sciences, University of California, Los Angeles, Los Angeles, CA, United States

**Keywords:** attachment, executive function, adolescence, antisocial behavior, mothering

## Abstract

Executive functions are considered essential for effective navigation in the social world. Parental responsiveness is a critical ingredient for normative social development and, as such, may be connected with the development of executive functions. Disruption of this development may, in turn, lead to maladaptive and antisocial behaviors. The purpose of this study was to evaluate the nature of the connections among perceived patterns of caregiving experienced in childhood, executive functions, and antisocial behaviors in at-risk adolescents. Seventy-one adolescent boys were recruited from two high-schools for adolescents who were not deemed suitable for regular schooling due to behavioral and emotional issues. Executive functions were tested using a computer-administered neuropsychological battery (CANTAB), and maternal parenting experiences and antisocial behaviors were assessed using retrospective and current questionnaires. Structural equation modeling (SEM) approach was employed to examine whether executive functions mediated the relationship between children's perceived patterns of maternal care and subsequent development of antisocial behaviors. Although maternal care had a significant direct effect on executive function (standardized coefficient = .49, *p* = .03) and antisocial behavior (standardized coefficient = .53, *p* = .05), SEM demonstrated no mediating relationships among these variables. Instead, maternal care predicted unique variance in both executive functions (standardized coefficient = .61, *p* = .02) and antisocial behavior (standardized coefficient = .51, *p* = .05). This study suggests a link between the experience of childhood caregiving and adolescent executive functions and delinquency and highlights the importance of early parenting interventions to aid executive function development. Such early interventions could potentially enhance long-term pro-social behavior.

## Introduction

At-risk youth outside of mainstream educational establishments have difficulty maintaining educational achievements (1) and are at a greater risk of a host of social difficulties (2). Youth who have been classified as “at-risk” demonstrate poorer executive functions, such as planning and monitoring, as compared to a community based control sample (3), and it is this specific element of cognitive functions that is widely related to the externalizing behaviors in children, youth, and adults (4).

Executive functions guide, direct, and manage cognitive, emotional, and behavioral functions (5). Although distinct categorization is contested, executive functions can be subdivided into shifting (switching flexibly between tasks or mental sets), updating (constant monitoring and rapid addition/deletion of working-memory contents), and inhibiting behavior (6). A similar, developmental, framework subdivides executive functions into inhibitory control (inhibition and selective attention), working memory, cognitive flexibility, and higher order executive functions (such as planning and reasoning), each with a slightly different developmental trajectory (7, 8). Executive functions have been related to a multitude of critical childhood achievements, such as school success (9), social competence (10), and emotional expression and experience (11). Conversely, deficits in executive functions, such as reduced inhibition, have been linked to poorer health, lower wealth, and greater involvement in crime (12).

Given the predominance of post-natal development in the pre-frontal lobe, the brain area most extensively related to executive functions, their development is highly susceptible to environmental influences (13), such as socioeconomic status (14) and early life stresses and traumas (15–17). Among these influences, maternal care seems to be especially relevant, given its pervasive role throughout child development. Maternal care typically includes sensitivity, affection, emotional warmth, empathy, and closeness (18). High quality maternal care is typically contrasted with absent or affectionless parenting, as well as low levels of overprotection (controlling or constraining behaviors), to provide optimal parenting (18). Longitudinal studies have shown that parental responsiveness and sensitivity to their infants have been related to improved executive function in their children in the years following initial testing (19). Similarly, parental ability to scaffold their children's learning and verbally guide problem solving at age two has been shown to predict increased executive functions at age four (20). Furthermore, the ability to fluidly move between closeness and exploration states, in addition to increased sensitivity to the child's need for help (a key “care” characteristic), as well as the ability of the parent to predict the child's needs at ages one and two, have been related to the executive functions of working memory, set shifting, and inhibitory control (13). The authors hypothesized that when children felt more secure to explore new environments, due to the reduced stress provided by secure and caring parenting, they were then freer to develop executive functions through gradual exploration.

Despite their potential connection, few published studies to date have examined the relationship between maternal care and adolescent executive functions. It is possible that the same maternal care characteristics that promote executive functions in early years will continue to be influential in later, adolescent development. As outlined above, the maternal care characteristics associated with optimal executive functions in children include warm, sensitive parenting, autonomy support, and clear and enforced boundaries (21, 22). Furthermore, low and unstable trajectories of the development of executive functions have been associated with increased externalizing behaviors, delinquency, and these deficits have been identified starting in early childhood (23).

In addition to frequently highlighted delinquency risk factors, such as suffering from parental separation (24), maltreatment, and harsh parenting (25, 26), there is some evidence that withdrawn maternal communication (27), or lability between levels of warmth displayed by mothers (28), is also associated with a significantly higher risk for antisocial or delinquent behaviors in adolescents. These results are coherent with the fluctuating detachment seen in depressed mothers, which is linked to later antisocial behavior in offspring (29, 30). Thus, it appears that the lack of caring parenting, or stability in caring parenting, may play an important role in increasing the risk of delinquency and antisocial behaviors.

**Figure 1:**
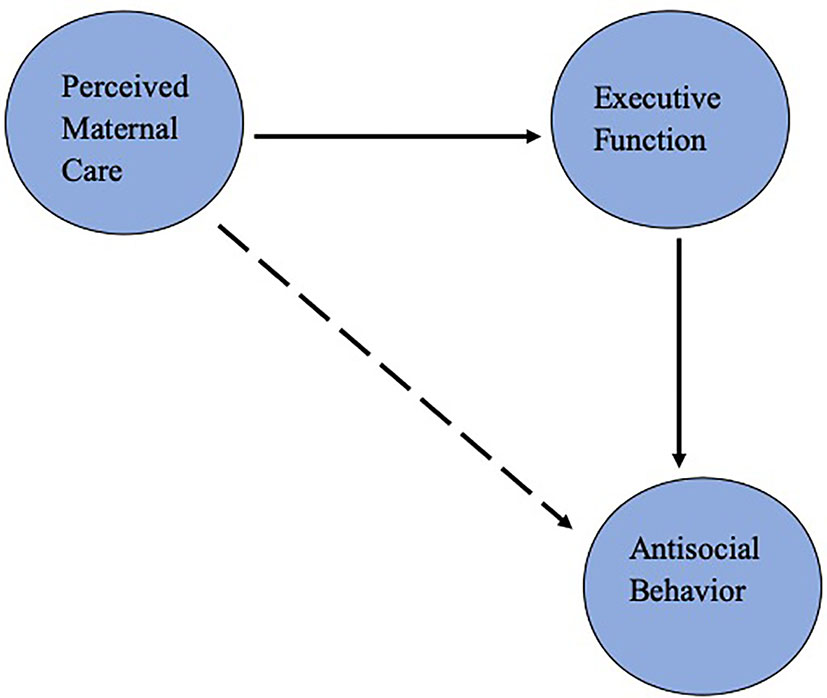
Schematic representation of the predicted mediation model. Solid arrows represent direct effects, and dashed arrow represents indirect effect.

In the present study, we examined the connection among the adolescent's perception of maternal care during their childhood, executive functions, and antisocial behavior. Given the relationship between executive functions and aggression, it has been suggested that deficits in executive functions may mediate the link between poor maternal care and aggression (31). To examine this proposal, a sample of at-risk adolescents living in low socioeconomic areas was recruited, given the increased risk of poorer executive functions in this population (3). We hypothesized a mediation model, in which executive functions would mediate the relationship between perceived maternal care and antisocial behavior. A schematic representation of this model is presented in **Figure 1**. To test this hypothesis, we utilized the structural equation modeling (SEM) approach. Conceptually, this approach is analogous to a combination of confirmatory factor analysis and multiple regression, as it allows the assessment of a common variance between latent variables and their indicators, as well as the variance or covariance among latent variables, within a theoretical framework. Thus, we were able to assess direct, as well as mediating, effects of the variables of interest on outcome.

## Methods

### Participants

The present study included 71 boys from 9^th^ through to 12^th^ grade, recruited into a prospective intervention study, examining the efficacy of martial arts training in at-risk youth. Given the substantial over-representation of boys at these institutions, reflecting the nature of these educational systems in dealing with severe externalizing behavioral problems, only boys were included to avoid potential selection bias. Participants were all students at two schools for at-risk youth in Israel. The schools are located in low socioeconomic areas: San Martin, Jerusalem, and Ramle. San Martin is an area of high immigration and purpose-built temporary accommodation and Ramle is home to Israel's largest prison and one of the highest crime rates in Israel. All participants were in regular high school matriculation classes (were not in the additional learning disabilities classes) but all had specific educational needs (ranging from disruptive behavior to nonattendance at previous educational establishments due to behavioral issues). Children are enrolled in these schools as a “last-resort” attempt to keep them in the general education system. The research was approved by the Institutional Review Board at Bar-Ilan University, the Israel Ministry of Education Ethics committee, and the Helsinki Ethics Committee of Hadassah Hospital in Jerusalem, as required by the Ministry of Health.

The boys ranged in age from 14 to 18.5 years (M = 15.8, SD = 1.04). Eighteen participants (26%) came from single parent families (living with mother). Twenty-one participants had a reported diagnosis of ADHD, nine were prescribed Ritalin, and four of these took Ritalin during the testing period. Given the prevalence of reported and un-reported ADHD within a sample of at-risk youth, it was decided to not exclude participants based on reported diagnoses of ADHD, although analyses were performed both with and without the four medicated participants with no significant difference. One boy admitted taking recreational drugs, and 14 admitted drinking alcohol during the previous week. Among 74% of participants, neither parent obtained higher education, and 38% participants were the children of immigrants. Parents of these children had a range of professions, with the most common for mothers was cleaning or household help and for fathers working in the service industry.

Baseline data were collected during the first weeks of the school year, using iPADs installed with the CANTAB cognitive assessment software (32) and questionnaires directly inputted into the Qualtrics research and production software. Data was anonymized with unique codes, and only the lead researcher had access to full coding information. All undergraduate level research assistants were trained in the testing procedures by the leading researcher.

## Measures

### Questionnaires

#### The Parental Bonding Instrument (PBI)

The PBI measures the perception of parenting during the course of the respondent's childhood (18). It shows long term stability over a 20-year period and is widely used in clinical and non-clinical studies to assess parenting styles (33, 34). The PBI has been compared to the Adult Attachment Interview (AAI) to good effect, although the significant association demonstrating the PBI's validity is only apparent for maternal, and not paternal PBI reports (35). The PBI questionnaire contains 25 self-report questions with 12 being rated for “care” (such as “spoke to me in a warm and friendly voice” and “frequently smiled at me”) and 13 for “overprotection” (such as “invaded my privacy” and “tried to control everything that I did”). Statements are scored (0–3) as how much the reflect the mother they remember during their first 16 years with 12 questions reverse scored. Given that the PBI is significantly correlated with AAI for maternal and not paternal reports and that the mother is typically the main caretaker, only the “mother” version of the questionnaire was used. Cut off scores (high care above 27 and high overprotection above 13.5) were based on previous validation studies (33, 36).

#### Delinquency and Aggression Factors of the Child Behavior Checklist, Youth Self Report

The Youth Self Report (37) is a widely used questionnaire for a range of child behavior issues (38) with participants indicating if each behavior is not true of them (1), sometimes true (2), or very true (3), reflective of their behavior in the past four months. The delinquency and aggression factors (30 questions) were extracted and translated and then checked by two independent mother tongue Hebrew-English researchers for accuracy. Statements of behaviors included, “I destroy a lot of my things” and “I am stubborn.” Additionally we added three questions from the Aggression Scale (39) to determine how often during the previous week the participants carried out acts of physical aggression. Questions include, “during the past week how many times did you hit or punch someone” and they are scored from 0 (0) to 6+ (6) times. Analyses were performed using raw scores.

### Cognitive Measures

All computerized neuropsychological measures were administered using the CANTAB cognitive assessment software (32). Participants were tested using the executive function battery, which included the Multitasking Test (MTT), One Touch Stockings of Cambridge (OTS), Spatial Working Memory (SWM), and Rapid Visual Information Processing (RVP).

#### Multitasking Test (MTT)

The MTT measures participant's ability to manage conflicting information provided by the direction of an arrow and its location on the screen and to ignore task-irrelevant information. Each trial displays a cue at the top of the screen that indicates to the participant whether they have to select according to the “side on which the arrow appeared” or the “direction in which the arrow was pointing.” In some sections of the task this rule is consistent across trials (single task) while in others it may change from trial to trial in a randomized order (multitasking). Using both rules in a flexible manner places a higher demand on cognition than using a single rule. Some trials display congruent stimuli (e.g., arrow on the right side pointing to the right) whereas other trials display incongruent stimuli, which require a higher cognitive demand (e.g., arrow on the right side of the screen pointing to the left). The mean incongruency cost (the difference between the mean latency of response, from stimulus appearance to button press, on the trials that were congruent versus the trials that were incongruent) from this test was used to demonstrate the participants level of inhibition (lower score equals higher inhibition). Given the test's key executive function properties, reaction time was calculated to generate speed of processing under significant cognitive demand.

#### One Touch Stockings of Cambridge (OTS)

The OTS is a test of executive functions, based upon the Tower of Hanoi test. It assesses both the spatial planning and the working memory subdomains. The participant is shown two displays containing three colored balls. The test administrator first demonstrates to the participant how to move the balls in the lower display to copy the pattern in the upper display and completes one demonstration problem, where the solution requires one move. The participant must then complete three further problems, one each requiring two moves, three moves, and four moves. Next the participant is shown further problems and must work out in their head how many moves the solutions require and then select the appropriate box at the bottom of the screen to indicate their response. The number of problems solved on the first choice was used to demonstrate the participants' general level of planning.

#### Spatial Working Memory (SWM)

The SWM provides a measure of strategy as well as working memory errors. The test begins with a number of colored squares (boxes) shown on the screen. The aim of this test is that by selecting the boxes and using a process of elimination, the participant should find one yellow “token” in each of a number of boxes and use them to fill up an empty column on the right-hand side of the screen. The number of boxes is gradually increased until a maximum of 12 boxes are shown for the participants to search. The color and position of the boxes used are changed from trial to trial to discourage the use of stereotyped search strategies. The participant's error score, when searching the median number of boxes (6), was used to demonstrate working memory under a moderate level of difficulty.

#### Rapid Visual Information Processing (RVP)

The RVP is a measure of sustained attention. A white box is shown in the center of the screen, with digits ranging from 2 to 9 appearing in a pseudo-random order, at the rate of 100 digits per minute. Participants are requested to detect target sequences of digits (for example, 2-4-6, 3-5-7, 4-6-8). When the participant sees the target sequence they must respond by selecting the button in the center of the screen as quickly as possible. The level of difficulty varies with either one- or three-target sequences that the participant must watch for at the same time. The total number of misses was used to demonstrate participants' level of selective attention.

### Data Analysis

All CANTAB output was converted to standard Z scores. Pearson bivariate correlations (two-tailed) were used to examine zero-order background correlations among the variables. T-tests were performed to examine potential group differences on variables that may influence measures of executive functions (e.g., participants who lived with single parents vs. those with both parents, took Ritalin, non-medicinal drugs, or alcohol).

Cognitive scores were chosen based on the suggested “key scores” generated from the CANTAB software and supported by previous research using the CANTAB software to examine executive functions (40). These scores represented the five key executive functions: behavioral inhibition, selective attention, flexibility, planning, and working memory. The latent variable “Maternal care” was indexed with two indicators: caring and overprotection. Finally, a latent variable of antisocial behavior was indexed with two indicators: delinquency report and aggression. The SEM technique was then used to examine the models hypothesized to explain the relationships among the latent variables and indicators (or measured variables), as well as the relationships among the latent variables.

The hypothesized models (both direct relationships and mediation) were tested using the Structural Equation Package EQS (41). This software reports many of the indices that have been described in the literature for evaluating model fit, including Bentler–Bonett normed fit index, Bentler–Bonett non-normed fit index, comparative fit index (CFI), Bollen fit index, McDonald fit index, Lisrel goodness of fit index (GFI), Lisrel adjusted GFI, root mean square residual (RMR), standardized RMR, and root mean-square error of approximation (RMSEA). As the fit indices were consistent in ranking the candidate models, we report in this paper the three commonly reported indices, the χ^2^, the CFI, and the RMSEA. A good fitting model is typically indicated by a non-significant χ^2^. However, because the χ^2^ is very sensitive to sample size, it often rejects good-fitting models (42). Therefore, the CFI and the RMSEA were also included (43). CFI values > 0.90 and RMSEA values < 0.10 typically indicate good model fit (44). The issue of missing data was addressed by first analyzing the data with list-wise deletion and repeating the analyses using pairwise deletion and maximum-likelihood expectation- maximization (45). As the pattern of results from the three methods for handling missing data was virtually identical, only the results obtained by using the maximum-likelihood expectation-maximization method are reported here. All standardized coefficients were interpreted based on the widely used conventions (i.e., 0.2 = small effect, 0.5 = medium effect, and 0.8 = large effect) (46).

## Results

Participants averaged six incidents of physical aggression in the previous week (*M* = 6.41, *SD* = 4.04), and their average score on the delinquency questionnaire was 44.06 (*SD* = 10.04), with scores ranging from 30 (indicating that they did not carry out any acts of delinquent behavior) to 88 (indicating that the majority of delinquent behaviors were endorsed in the past four months). They reported receiving a high level of caring behaviors from their mothers (*M* = 28.47, *SD* = 6.63, range = 12–36). However, when participants were grouped into those who experienced low care, according to a cut-off score of 27.0, over twice the number of participants were characterized as experiencing a low level of care from their mothers (47 low care, 22 high care). As in experience of caring, so too was there a wide range of experienced levels of overprotection, (cut off 13.5, *M* = 13.72, *SD* = 6.09, range = 0–30). Level of care experienced by the child was significantly related to measures of executive functions, including inhibition (*r* = −0.36, *p* < 0.01), speed of processing (*r* = −0.37, *p* < 0.01), and working memory (*r* = 0.32, *p* < 0.01). Pearson's bivariate correlations (two tailed) for the study variables are reported in **Table 1**.

**Table 1:** Pearson's Correlations among key variables.

	2	3	4	5	6	7	8	9
1 Speed of Processing	.30*	−.04	.27*	−.41**	−.37**	.13	.30*	.14
2 Selective Attention		.13	−.03	−.32**	−.09	.04	−.00	.17
3 Working Memory			−.05	−.20	.32**	−.15	−.24*	−.11
4 Inhibition				−.07	−.36**	.19	.07	.08
5 Planning					.20	−.07	−.02	−.05
6 Caring						−.33**	−.12	−.20
7 Overprotection							.21	.34**
8 Delinquency								.42**
9 Aggression								

* = p < 0.05; ** = p < 0.01.

T-tests demonstrated no significant differences on executive function measures for those youth who lived with both parents or just one parent (all *p*'s > 0.2). There was no significant difference in whether they perceived their mothers as caring (*t*(66) = 1.23, *p* = 0.223) or overprotective, (*t*(66) = −1.06, *p* = 0.262), whether they lived with both parents or with their mothers. Similarly, there were no significant differences (all *p'*s > 0.05) in executive function scores based on medication taking (specifically Ritalin), alcohol use in the previous week, or drug use over the previous four months (likely reflecting the small variation of drug use among participants).

### Structural Equation Models

The models were estimated using maximum likelihood solution. The measured indices of care and overprotection loaded on the overall perceived maternal care factor, such that higher maternal care and lower maternal overprotection related to more positive outcomes. The basic model, hypothesizing a direct relationship between a factor of perceived maternal care and executive function, was tested first. The executive functions of processing speed, selective attention, behavioral inhibition, and planning, and working memory were entered into the model as a single executive function factor. However, working memory loaded poorly on the executive functions factor (standardized coefficient = 0.05, *p* > 0.05). To improve model fit, *post hoc* modifications were performed (42). Using the Lagrange multiplier and the Wald tests (42), as well as considering theoretical relevance, working memory was dropped from the executive functions latent variable. The resulting model provided a strong fit for the data.

**Figure 2:**
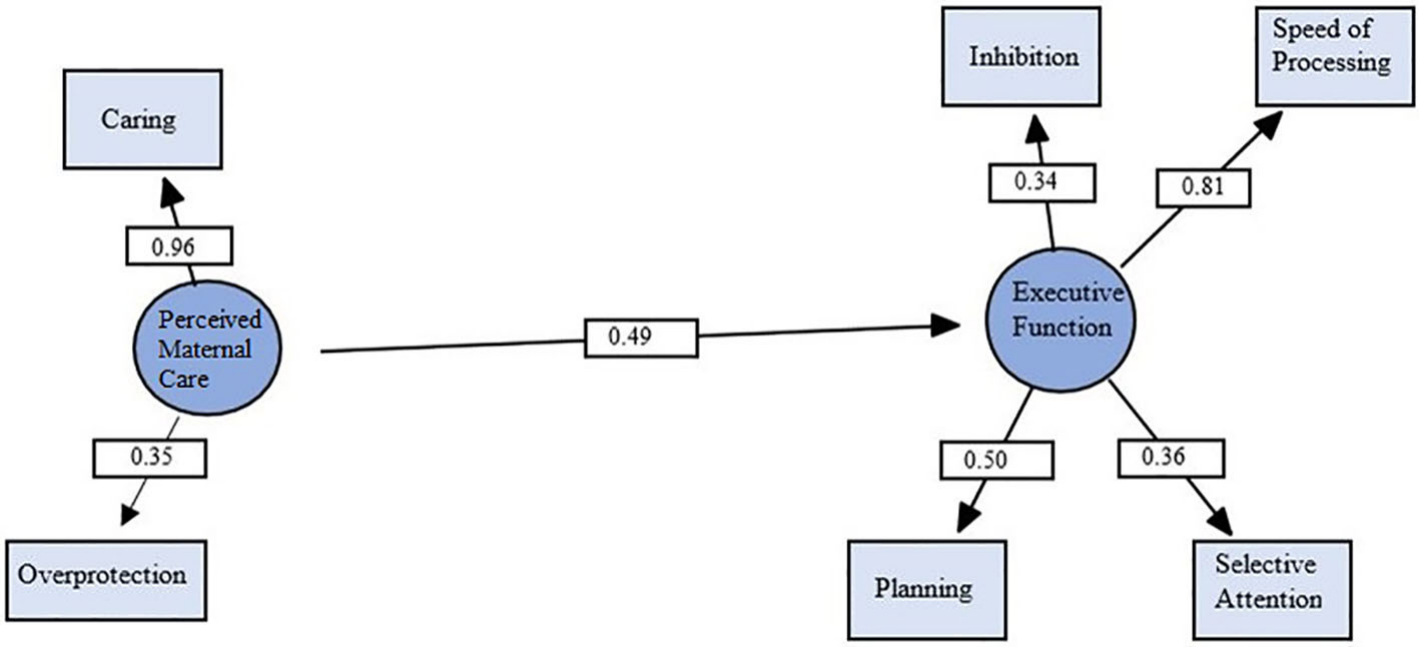
Structural equation model demonstrating relationship between perceived maternal care and executive functions. Circles represent latent variables, and rectangles represent measured variables. Values are standardized path coefficients. χ^2^ (8, N = 71) = 9.30, *p* = .32, CFI = .97, RMSEA = .05. *p* < .05 for all coefficients.

The independence model, testing whether the observed data fit the expected data, was rejected, χ^2^ (15, N = 71) = 54.76, *p* < 0.001. (The χ^2^ for the independence model should always be significant, indicating that there is a relationship among the variables.) The basic model provided a strong fit for the data. As can be seen in **Figure 2**, all indicators had moderate-to-high loadings on their respective latent variables and all were significant (*p* < 0.05). Importantly, the latent variable perceived maternal care (care and overprotection) had a significant direct effect on executive functions (standardized coefficient = 0.49, *p* = 0.028, medium effect size).

**Figure 3:**
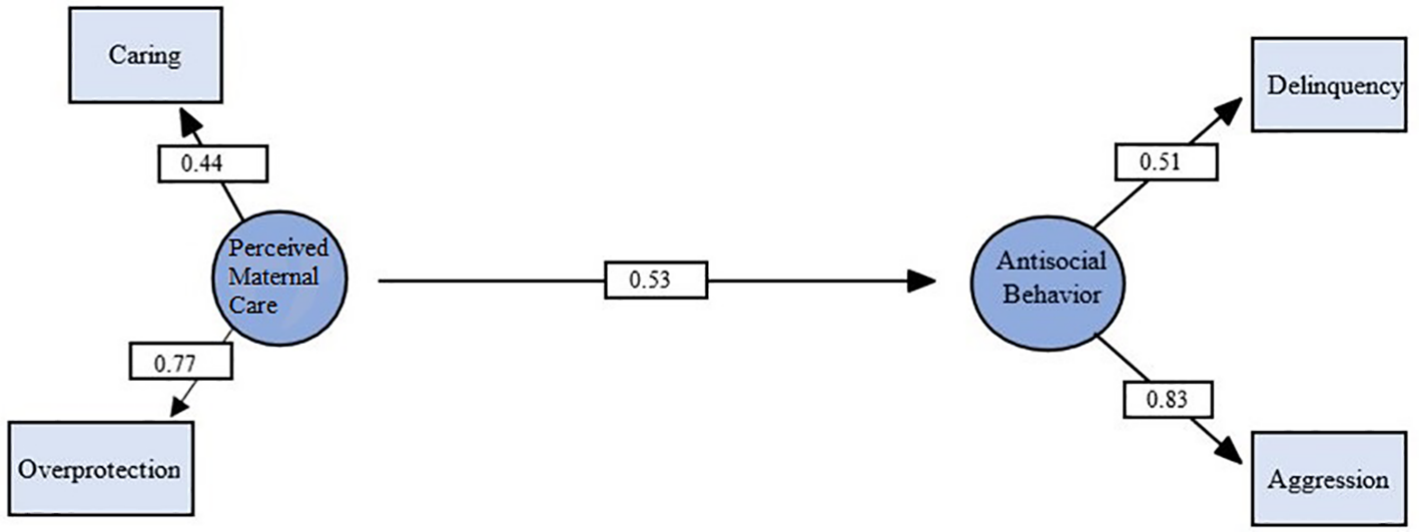
Structural equation model demonstrating relationship between perceived maternal care and antisocial behavior. Circles represent latent variables, and rectangles represent measured variables. Values are standardized path coefficients. χ^2^ (1, N = 71) = .002, *p* = .97, CFI = .99, RMSEA = .001. *p* < .01 for all coefficients.

Similarly, a model testing a direct connection between perceived maternal care and antisocial behavior provided a strong fit for the data. The independence model was again readily rejected, χ^2^ (6, N = 71) = 31.49, *p* < 0.001. As can be seen in **Figure 3**, all indicators had moderate-to-high loadings on their respective latent variables and all were significant (*p* < 0.05). The latent variable perceived maternal care had a significant direct effect on antisocial behavior (standardized coefficient = 0.53, *p* = 0.010, medium effect size).

**Figure 4:**
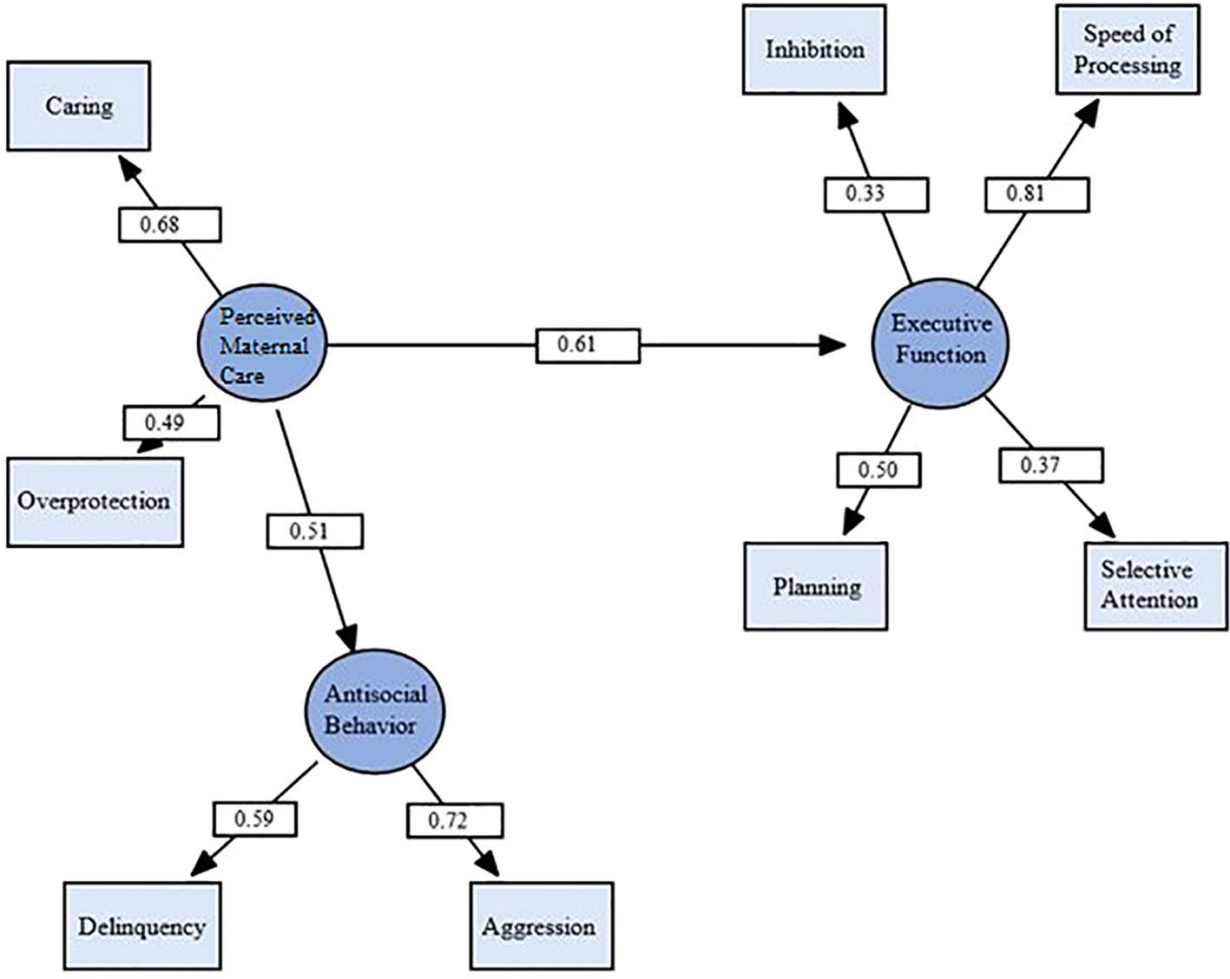
Structural equation model testing the effect of perceived maternal care on executive function and antisocial behavior. Circles represent latent variables, and rectangles represent measured variables. Values are standardized path coefficients. χ^2^ (18, N = 71) = 23.47, *p* = .17, CFI = .91, RMSEA = .07. *p* < .05 for all coefficients.

Subsequently, in the mediation model, both the direct path from perceived maternal care to antisocial behavior and the indirect path through executive function were evaluated. There was no mediation demonstrated, as both the direct path from executive functions to antisocial behaviors (standardized coefficient = 0.15) and the indirect path from perceived maternal care to antisocial behaviors through executive functions (standardized coefficient = 0.09) were not significant (*p* > 0.05). Instead, we found that perceived maternal care accounted for significant and independent variance in both executive functions and antisocial behaviors. The final model is presented in **Figure 4**. The independence model was again readily rejected, χ^2^ (28, N = 71) = 88.31, *p* < 0.001. As can be seen in **Figure 4**, the final model provided a good fit for the data, and all indicators were significantly related to their respective latent variables (*p* < 0.05). Executive function was significantly predicted by perceived maternal care (standardized coefficient = 0.61, *p* = 0.016, medium to large effect size), as was antisocial behavior (standardized coefficient = 0.51, *p* = 0.046, medium effect size).

## Discussion

The current findings support the importance of the experience of caring maternal parenting (a high level of experienced care and lower level of overprotection) in the development of executive functions and antisocial behaviors. Additionally, they highlight the increased level of perceived low maternal care amongst at-risk adolescents. Well-adjusted adolescents are typically characterized by a high level of care and low level of overprotection (47), but in the current sample of at-risk adolescents, over two thirds perceived low levels of maternal care. This puts adolescents at risk of a range of psychopathologies (33, 36, 48) and socio-emotional problems (47).

We hypothesized that secure maternal care, which has been demonstrated in previous studies to be predictive of young children's executive function development (20), would be replicated as a predictor in an adolescent sample. Key characteristics of parenting styles that were demonstrated to relate to child executive functions included autonomy support and maternal sensitivity (21). We were able to assess adolescents' retrospective views to what extent they felt their mothers showed these characteristics, which were encompassed within the two key maternal variables of “care” and “overprotection.” As predicted, there was a significant relationship between the majority of executive function variables (we removed working memory, as outlined below) and the perceived level of maternal care experienced by at-risk adolescents throughout their childhood. Maternal care was judged based on how much adolescents felt their mothers were affectionate, understanding of their experiences, and how invested they were in their children's wellbeing. It included the perception of how much the adolescents felt their mothers could help them regulate their emotions. Maternal overprotection was judged based on what extent the adolescents felt they weren't encouraged to have autonomy and self-direction. Greater perceived maternal care and lower perceived overprotection experienced by the at-risk adolescents in this study was significantly related to greater performance on executive function tests.

Whereas our model for executive function revealed significant loadings for selective attention, inhibition, planning, and speed of processing, all sharing common variance with maternal care, working memory did not have a significant loading on the executive function factor. Instead, working memory demonstrated an independent relationship to maternal care, separate from the general executive function factor. This division of speed of processing, inhibition, selective attention, and planning as key executive functions, with working memory as a somewhat independent cognitive function, concurs with a narrower view of higher executive functions as demonstrating differential trajectories and the findings showing a closer relationship of working memory with general cognitive intelligence rather than executive functions (49).

Working memory appears to have a different trajectory of development than other executive functions (7, 50) and has been recently categorized in a two factor model (working memory and inhibition) used to capture overall executive abilities (51). It should also be noted that the measure of spatial working memory used in the present study may index a more basic memory process than is often captured by higher executive functions (52). Although our findings give some credence to a separation of previously united executive functions, the unique nature of our sample and the limited characterization of working memory leave ample room for future research with normative samples and broader assessments of working memory to further evaluate these results.

In addition to our findings in relation to executive functions, so too were we able to build a model demonstrating a strong and direct connection between the factor of perceived maternal care and that of antisocial behavior. This model builds on the realm of research documenting the predictive nature of poor parental attachment and later criminogenic behavior. Beginning with Bowlby's pioneering study (24) and Farrington's longitudinal study of delinquency in youth (53, 54), erratic, hostile parenting has been long associated with antisocial behavior. Poor parental management has been repeatedly demonstrated to be a leading factor in antisocial and delinquent behaviors (55). However, the aforementioned studies assess severe parenting abnormalities, such as violence in the home, removal from the home (55), parental neglect, parental drug use, sexual abuse (25), and severe poverty (56). Where subtle changes in parental warmth or hostility have been studied, changes in risk taking behaviors have been observed during periods of especially high or low hostility, and this lability was related to greater delinquency in girls but not in boys (28). Our study, using only at-risk boys, found that when at-risk boys reported higher levels of caring behaviors and lower levels of overprotective behaviors from their mothers throughout their childhood, they were less likely to display antisocial behaviors. Despite not being able to participate in traditional educational establishments and thus already displaying higher levels of risk towards antisocial behaviors (57), personal experience of maternal care still demonstrated a strong ability to predict greater antisocial behavior and differentiate between at-risk boys.

Recent studies have highlighted the move towards “enrichment parenting,” namely, stimulating and fostering the child's future academic success (58). Enrichment parenting includes the sending of children to multiple stimulating after-school activities, utilizing educational media and getting a “head-start” on learning prior to school level education. Indeed, Orri and colleagues (59) argued that in low socioeconomic situations, increased use of childcare during early childhood was actually helpful in reducing antisocial behavior in adolescence, and after-school clubs have been demonstrated to increase executive functions (60). In the present study, rather than researching intervention strategies or severe parental abnormalities, we assessed a subtle style of parenting, suggesting some potential for enrichment and education. Rather than delegating parental input, the current study lends support to early parenting interventions to facilitate caring and nurturing behaviors.

Contrary to our prediction, we did not find a mediating role of executive functions between perceived maternal care and antisocial behavior. Instead, the factor of perceived maternal care predicted unique variance in both executive functions and antisocial behavior. It is possible that other factors, such as parenting style and early educational experiences, not included in the present study, may underlie these relationships (61). Nonetheless, the close relationship between maternal care with executive functions and antisocial behaviors in at-risk adolescents underscores the importance of early efforts toward secure attachment and mothering style. Aiding parents to utilize thoughtful and caring parenting strategies which help children regulate, validate, and moderate their emotional experiences and feel secure and cared for, may be essential to educational and behavioral achievement.

A number of limitations of the present study need to be acknowledged. First, the self-report measure of attachment used in the current study mostly reflects participants' view of their mothers' care taking, which could be discrepant from the mothers' actual care taking style. Unfortunately, these type of school settings are often characterized by severe familial discord, with parents showing little engagement in their children's education. Despite repeated attempts, we were unable to obtain corroborating data on attachment from parents. To deal with this issue, we employed a standardized measure to assess the perception of parenting during the course of the respondent's childhood (18), which is widely used in clinical and non-clinical studies to assess parenting styles with demonstrated long-term stability over a 20-year period (33, 34). Nonetheless, it would be informative to conduct additional studies evaluating the degree of consistency in reporting on measures of attachment between parents and children in similar populations.

Another limitation was the lack of information on paternal care. Due to traditional maternal caring roles characterizing this sample and the overwhelming majority of maternal (as compared with paternal) single-parent households, the present study focused on maternal, rather than paternal, care. However, it is possible that factors related to paternal attachment and care may contribute unique variance to the development of executive functions and antisocial tendencies. Obtaining information directly from parents was extremely difficult in the sample population, as many parents had little contact with the schools (only around 10% of parents attended the opening school event) and the level of Hebrew or English language literacy was poor. It could potentially be beneficial, in future research, to utilize home visits for obtaining parental reports, as well as observational data.

Finally, this study utilized a cross-sectional design and, as such, the present findings should be considered preliminary. Data collected in this cross-sectional manner could have resulted in some bias inherent in retrospective reporting. Future research could benefit from addressing similar questions using a controlled, longitudinal approach. Specifically, it would be informative to examine whether the pattern of findings among early maternal care, executive functions, and antisocial behavior demonstrated in the present model would remain in studies that follow children over time. Additionally, as the present sample included only at-risk adolescents, it remains unclear whether these findings are generalizable to other adolescent populations and remain when a control sample of “high care” adolescents are studied compared to “low care” adolescents. It would therefore be informative to test similar models in adolescents within normative school settings. Despite these limitations, the lack of ability to function in a traditional school setting and the high level of delinquency and aggressive behaviors, combined with the overall low level of care perceived, corroborates the predictive value of maternal care on antisocial behavior, as demonstrated in the final model.

The present findings thus demonstrate the impact of maternal care on executive functions and antisocial behavior in at-risk adolescents. Prior research has demonstrated the impact of sensitivity and attachment from mother to child in the development of early executive functions in preschoolers (13, 62). The present study extends this research to show the impact of maternal care on executive functions in adolescents. Despite the importance of maternal care on improving cognitive function and reducing delinquency, our data present the sad reality of the low level of caring experienced by at-risk youth. This study highlights the importance of implementing early parenting interventions in efforts to boost later educational and social success in at-risk youth. In areas where there are high levels of socio-economic risk, it would be critical to work with parents to enhance their sensitivity and level of caring demonstrated to their children. This can be implemented through interventions, such as parenting programs, information evenings, mother's helper volunteers (e.g., students or pensioners), or early interventions provided by trained clinicians. Further research is needed to determine which early-year parenting interventions are most successful in improving later executive function development and reducing antisocial behavior in older children and adolescents.

## Data Availability Statement

The datasets generated for this study are available on request to the corresponding author.

## Ethics Statement

The studies involving human participants were reviewed and approved by Institutional Review Board at Bar-Ilan University, Israel Ministry of Education Ethics Committee and Helsinki Ethics Committee of Hadassah Hospital in Jerusalem. Written informed consent to participate in this study was provided by the participants' legal guardian/next of kin.

## Author Contributions

AH-G and YR conceived the study, participated in the design and coordination of the study, and performed analysis and interpretation of data. AH-G also performed data collection and oversaw research assistants. BL assisted in data collection and analysis and manuscript preparation. RF critically assessed study conception and manuscript preparation.

## Funding

This research was supported by the Ministry of Science, Technology & Space, Israel (Grant #3-13631 to YR and RF). This study was carried in the course of the PhD research conducted by AHG at Bar-Ilan University, Ramat Gan, Israel. AH-G is a recipient of the Presidential Scholarship Award from Bar-Ilan University.

## Conflict of Interest

The authors declare that the research was conducted in the absence of any commercial or financial relationships that could be construed as a potential conflict of interest.
